# Musculoskeletal fitness: relative handgrip strength and vertical jump power from 10 to 18 years old

**DOI:** 10.3389/fped.2024.1207609

**Published:** 2024-01-25

**Authors:** Abel S. Correia, Vera Zymbal, Fátima Baptista

**Affiliations:** ^1^ CIPER, Faculdade de Motricidade Humana, Universidade de Lisboa, Lisbon, Portugal; ^2^ Instituto Politécnico de Setúbal, Escola Superior de Saúde, Setúbal, Portugal

**Keywords:** musculoskeletal health, handgrip strength, jump power, mechanography, reference standards

## Abstract

**Background:**

There is an increasing consensus on the relevance of musculoskeletal fitness for health throughout the life cycle, requiring evaluation approaches and description of results capable of characterizing different age groups and body sizes. This study aimed to describe the musculoskeletal fitness of young Portuguese people aged 10–18 through handgrip strength (HGS) and vertical jump power (VJP) and investigate differences between the sexes.

**Methods:**

The sample included 736 participants (359 girls recruited from schools. HGS (kg) was assessed using a handheld dynamometer, and VJP (W) was assessed using a force platform; both measurements were standardized for body mass.

**Results:**

Higher HGS and VJP were observed in boys than in girls from 13 years old (13 years: *p* ≤ 0.05; 14–18 years: *p* ≤ 0.001), with no significant differences before this age. The percentile distributions of HGS and VJP are described for each sex using the lambda, mu, sigma (LMS) method. The pattern of development of these variables as a function of age is presented.

**Conclusions:**

Handgrip strength and vertical jump power show differences between the sexes from 13 years of age and similar trajectories to populations in other countries in the same age group.

## 1 Introduction

Musculoskeletal fitness refers to the muscle's ability to perform activities that require maximum levels of muscle contraction against a load (muscle force) or that require submaximal muscle contractions (repeated or sustained) over a prolonged period (strength resistance) or that require the production of force in a short period (muscle power) (1).

In adults, musculoskeletal fitness has been associated with nutritional status (2), disease and cancer mortality risk, and all-cause mortality (3, 4). In older people, musculoskeletal fitness is a biomarker of sarcopenia, frailty, and risk of falling and a predictor of the risk of physical disability, cognitive impairment, institutionalization, hospitalization, and mortality (5, 6). In children and adolescents, musculoskeletal fitness is a determinant of health in general and cardiovascular, metabolic, bone, and mental health in particular (7, 8), but also neuromotor development (9) and associated with future health benefits (10). Musculoskeletal fitness has also been recently proposed as a biomarker of pediatric sarcopenia risk, although health-referenced values should be used with caution due to potential population differences, particularly in body size (11).

Musculoskeletal fitness can be assessed through several approaches: assessment of strength, resistance, or power, assessment of upper limbs, lower limbs, or trunk (or muscle groups in these body regions), and evaluation with field or clinical/laboratory tests (12). Considering the objective and resources, assessment approaches tend to differ depending on whether they are athletic or non-athletic populations, age groups, and respective occupational contexts: youth/school or club, adults/work or gym, elderly people/community, and nursing homes. Handgrip strength at the upper limbs (kg) and the power of vertical jump at the lower limbs (W) are among the most common assessment approaches in different age groups and contexts, thus allowing comparative analyses of muscular fitness throughout the life cycle. These are assessments with attested measurement properties (13), with a health-related discriminatory ability (14) and recommended by the World Health Organization (15).

The impairment of health in different population groups, or growth and development in the youngest and intrinsic capacity in the older, can only be identified if there are reference values for musculoskeletal fitness predictive of these impairments (16–18). Several studies on children and adolescents have been published for this purpose in different regions of the world, especially for handgrip strength: Belgium (19), Sweden (9), South Korea (20–22), Great Britain (23, 24), Netherlands (25, 26), Australia (27), Brazil (28, 29), Korea (30), United States of America (31–33), Canada (34), Colombia (35), Chile (36, 37), Saudi Arabia (38), Turkey (39), Iran (40), Serbia (41), and Galicia, Spain (42).

Considering the requirement of activities of daily living, from the most basic (self-care activities) to the most advanced (personal or professional enrichment activities) with greater ambulatory demand, more and more attention has been devoted to the muscle power of the lower limbs of the older people (43), but also the younger ones (44). Reference values for vertical jump power in children and adolescents have been published with samples from Germany (43), the Czech Republic (44), and Canada (45). Many more studies have expressed vertical jump performance through distance measurements (cm), which are easier to obtain (46, 47). Jump height is a marker of muscle power, and the two variables are strongly correlated. However, identical jump heights in two subjects with different body masses do not reveal the same muscle power. Thus, it is preferable to express musculoskeletal fitness through vertical jump power (48) adjusted to body mass or other body composition/size markers to classify an individual within a group or diagnose muscle weakness.

Given the importance of musculoskeletal fitness in children and adolescents, it is necessary to describe normative values and identify cut-off values below which health, growth, or development may be compromised or limited (health-referenced values) (49). The primary aim of this study was to describe the musculoskeletal fitness of young Portuguese people aged 10–18 years. The secondary aims were to investigate sex differences in musculoskeletal fitness and to compare Portuguese youth's musculoskeletal fitness with normative values from other countries. The existence of discrepancies between values can hinder the widespread adoption of reference values for health risks (11, 50). For comparison purposes, we considered the handgrip strength at the level of the upper limbs and the jumping power at the lower limbs adjusted for body mass, using procedures similar to those implemented to describe musculoskeletal fitness in young people from other countries.

## 2 Methods

### 2.1 Subjects

The sample consisted of 736 participants (359 girls and 377 boys, 96% white) aged between 10 and 18, from the 4th to the 12th grade. Participants were recruited from 6 schools as part of two different studies: the Lisbon Study to characterize the Physical Fitness and Physical Activity of its Citizens (207 participants out of 270 students recruited from 2 schools, 76%) (51, 52), and the Lisbon Study for the Development and Evaluation of Musculoskeletal Fitness Standards in Youth (529 participants out of 720 students recruited from 4 schools, 73%) (49). Data were collected between March 2017 and May 2018 by a group of evaluators trained to standardize the tests performed and thus minimize error. Participants had to be aged 10 to 18 years and able to perform the physical tests to be included in the study. Informed consent was obtained from all parents, including those of participants aged 18, to participate in the study.

### 2.2 Body mass, body height, and somatic maturity

Body mass (kg) was determined with an electronic scale (Seca, Hamburg, Germany). Height was assessed while standing with a stadiometer to the nearest 0.1 cm. All participants were evaluated barefoot and with their heads positioned according to the Frankfurt plan (53). The respective values result from the average of two measurements in each variable. The body mass index (BMI) was calculated from the body mass/height ratio (kg/m^2^). Somatic maturity was estimated as the years of distance, positive or negative, from the age of peak height velocity (PHV) using sex-specific prediction equations that include age and height (54).

### 2.3 Musculoskeletal fitness

Musculoskeletal fitness was assessed in the upper limbs using a handgrip strength test (Jamar, Lafayette, IN, USA) and in the lower limbs using countermovement vertical jump power on a force platform (Leonardo Mechanograph, Novotec Medical GmbH, Germany). The musculoskeletal fitness of the upper limbs was expressed in kilograms (kg), and in the lower limbs, it was expressed in watts (W).

The handgrip strength assessment was performed twice on each hand, alternately with 30–60 s of rest between attempts, in the standing position with the supports aligned with the hips, with the dynamometer away from the body pointing downwards and aligned with the forearm at the thigh level. The maximum isometric contraction was maintained for approximately 2 s. To evaluate the vertical jump power, the participants performed a practice jump and two test jumps with 30 s of rest between attempts. For this purpose, the participants assumed a jumping position with their hands on their waist and their feet hip-width apart. The best result in each of the musculoskeletal tests was considered. Other authors describe the validity and reproducibility of these tests elsewhere (55, 56).

### 2.4 Health condition

The general state of health was evaluated through a questionnaire given to the guardians regarding diseases, medication use, and bone fractures. Nine participants reported cardiovascular problems (murmur, arrhythmia, and peripheral vascular), 28 participants reported asthma and 115 participants reported a prior bone fracture. None of these 152 participants reported any limitations to the physical performance tests and were included in the study. No differences in handgrip strength and vertical jump power were observed between these participants and the rest (*p* > 0.05).

### 2.5 Statistical analysis

Data referring to the sample description are presented as mean and standard deviation, calculated using the SPSS program (Version 25 for Windows; IBM, New York, USA). For data analysis, we proceeded: (a) to characterize the sample separated by sex and using the original values of the variables (absolute values); (b) the use of the lambda, mu, sigma (LMS) method through the LMS ChartMaker Light (Version 2.5, The Institute of Child Health, London, UK) to construct LMS tables and percentile graphs (3, 10, 25, 50, 75, 90 and 97) by age for both sexes (relative values, standardized for body mass); (c) the analysis of the differences in the variables between boys and girls, separately for each age (or somatic maturity) group through *t*-tests for independent samples, with verification of the prerequisites relating to normality and homogeneity.

For the constitution of age groups, the midpoint of the respective group was considered, namely the 10-year-old group, which included all children aged between 9.50 and 10.49 years, and so forth. The annual gains in handgrip strength and vertical jump power per kg of body mass were estimated as the difference between each age concerning the immediately previous age. The results of our sample were also compared (graphically) with those of samples from studies carried out in other countries for handgrip strength (35, 36, 57) and vertical jump power (44, 45).

## 3 Results

The sample consisted of 736 participants, of which 359 were girls and 377 were boys aged between 10 and 18, according to the age grouping shown in Table 1.

**Table 1:** Number of participants (10–18 years old) in each age group^a^.

Age (years)	Total (*n*)	Girls (*n*)	Boys (*n*)
10	103	58	45
11	94	47	47
12	87	39	48
13	79	34	45
14	101	57	44
15	91	50	41
16	74	29	45
17	80	33	47
18	27	12	15
TOTAL	736	359	377

^a^ The 10-year-old group includes children between 9.50 and 10.49 years old, and so on.

The characterization of the sample, namely, body mass, body height, and BMI are presented in Table 2, separately for boys and girls.

**Table 2:** Characterization of the sample, expressed as the mean ± standard deviation.

Age (years)	Body mass (kg)	Body height (cm)	BMI (kg/m^2^)
Girls	** **	** **	** **
10	37.0 ± 7.1	142.2 ± 6.4	18.2 ± 2.7
11	42.3 ± 9.0	149.1 ± 6.4	18.9 ± 3.2
12	49.5 ± 7.1	154.9 ± 5.8	19.3 ± 2.5
13	50.8 ± 8.1	157.5 ± 6.4	20.4 ± 2.8
14	53.8 ± 8.9	160.7 ± 5.7	20.8 ± 3.7
15	54.9 ± 9.4	160.8 ± 6.9	21.2 ± 3.2
16	57.6 ± 7.2	162.5 ± 5.6	21.7 ± 2.3
17	57.6 ± 10.9	161.6 ± 7.0	22.0 ± 3.8
18	53.7 ± 6.5	160.1 ± 9.6	21.1 ± 4.0
Boys			
10	37.9 ± 9.0	142.9 ± 7.9	18.3 ± 3.1
11	39.7 ± 6.8	146.3 ± 6.4	18.4 ± 2.3
12	44.4 ± 10.3	153.3 ± 8.3	18.7 ± 2.9
13	50.0 ± 11.9	157.8 ± 8.2	19.8 ± 3.3
14	57.6 ± 10.2	167.7 ± 7.7	20.3 ± 2.4
15	58.7 ± 12.4	167.6 ± 9.6	21.1 ± 5.8
16	65.9 ± 8.9	174.6 ± 7.1	21.5 ± 2.4
17	65.5 ± 10.5	175.0 ± 6.4	21.5 ± 3.2
18	64.5 ± 8.3	176.1 ± 5.1	20.7 ± 2.3

**Figure 1:**
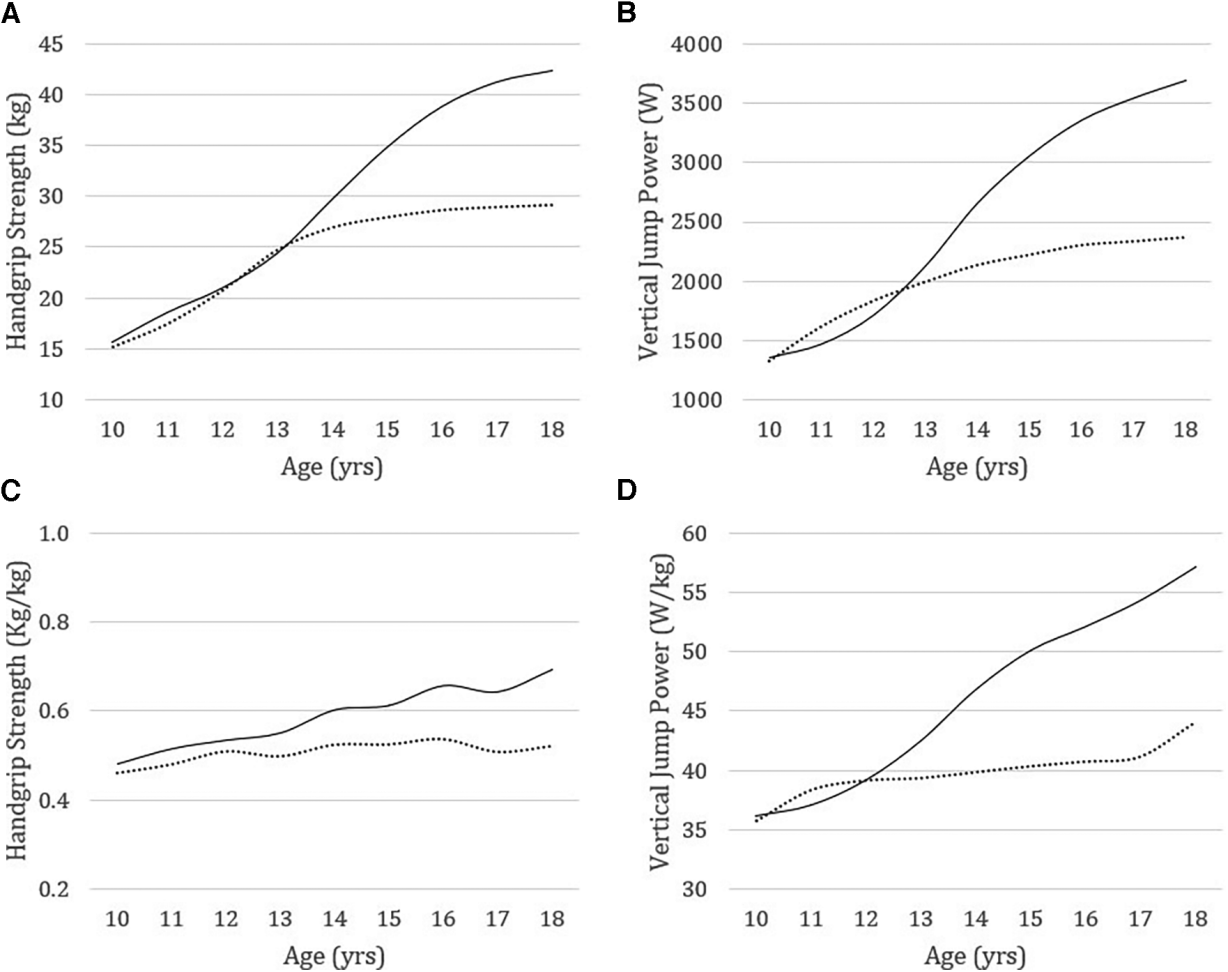
Handgrip strength and vertical jump power without (**A**,**B**) and with adjustment for body mass (kg) (**C**,**D**) in boys (solid line) and girls (dashed line) from 10 to 18 years of age.

**Figure 2:**
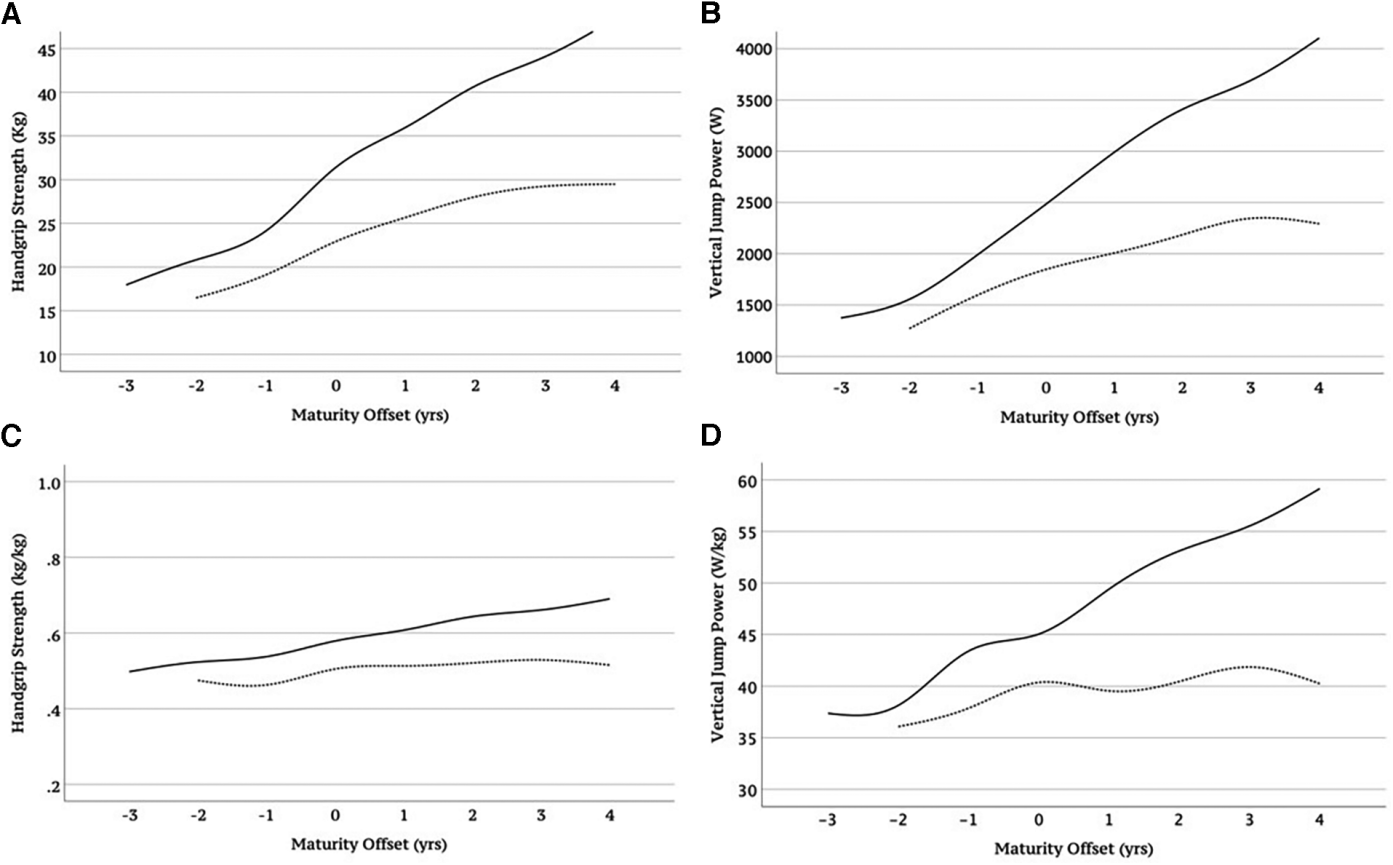
Handgrip strength and vertical jump power without (**A**,**B**) and with adjustment for body mass (kg) (**C**,**D**) in boys (solid line) and girls (dashed line) according to somatic maturity.

 Table 3 describes the results expressed as the mean and standard deviation of handgrip strength (kg) and vertical jump power (W), including the adjusted results for body mass (kg/kg, W/kg) according to sex and age group. Compared to girls, boys showed higher handgrip strength and jumping power from the age of 14 onwards (14–18 years: *p* ≤ 0.001). When musculoskeletal fitness is adjusted for body mass, differences between the sexes are evidenced from 13 years of age, both in handgrip strength (13 years: *p* = 0.01) and vertical jump power (13 years: *p* = 0.005), with no significant differences before these ages (Figure 1). When considering somatic maturity (distance in years to PHV), which in our sample ranged from −2 to 5 years in girls and from −3 to 4 years in boys, greater handgrip strength and vertical jump power (absolute and relative) were observed in boys than in girls (*p* < 0.001) except groups with somatic maturity of −2 years in which boys had greater relative vertical jump power (*p* < 0.05) but not relative handgrip strength (Figure 2).

**Table 3:** Mean ± standard deviation, mean difference and 95% confidence interval of the difference of handgrip strength and jumping power according to sex and age group without and with adjustment for body mass (kg).

Age	Girls	Boys	Mean Diff	95% CI	*P*	Girls	Boys	Mean Diff	95% CI Diff	*P*
	Handgrip Strength (kg)					Handgrip Strength/ (kg/kg)				
10	16.8 ± 3.2	17.9 ± 3.8	−1.1	(−2.5, 0.3)	0.110	0.46 ± 0.1	0.48 ± 0.1	−0.02	(−0.06, 0.29)	0.275
11	19.9 ± 3.6	20.0 ± 3.5	−0.1	(−1.5, 1.3)	0.883	0.48 ± 0.1	0.51 ± 0.0	−0.03	(−0.08, 0.01)	0.088
12	23.4 ± 3.7	23.0 ± 3.8	0.4	(−1.2, 2.0)	0.633	0.50 ± 0.0	0.53 ± 0.0	−0.03	(−0.07, 0.01)	0.197
13	25.1 ± 4.1	27.1 ± 6.1	−2.0	(−4.4, 0.5)	0.111	0.49 ± 0.0	0.55 ± 0.0	−0.06	(−0.09, −0.01)	0.014
14	27 ± 3.7	27.1 ± 6.1	−2.0	(−4.4, 0.5)	0.111	0.49 ± 0.0	0.55 ± 0.0	−0.06	(−0.09, −0.01)	0.014
14	27.9 ± 4.7	34.5 ± 8.3	−6.6	(−9.4, −3.8)	≤0.01	0.52 ± 0.0	0.60 ± 0.0	−0.08	(−0.12, −0.04)	≤0.001
15	28.4 ± 4.2	35.5 ± 7.6	−7.1	(−9.7, −4.4)	≤0.001	0.52 ± 0.0	0.61 ± 0.0	−0.09	(−0.13, −0.05)	≤0.001
16	30.5 ± 4.0	43.0 ± 8.2	−12.6	(−15.4, −9.7)	≤0.001	0.53 ± 0.0	0.65 ± 0.0	−0.12	(−0.17, −0.07)	≤0.001
17	28.9 ± 4.6	41.7 ± 7.0	−12.8	(−15.4, −10.2)	≤0.001	0.50 ± 0.0	0.64 ± 0.0	−0.14	(−0.18, −0.09)	≤0.001
18	27.9 ± 4.3	44.5 ± 6.2	−16.6	(−21.0, −12.3)	≤0.001	0.52 ± 0.0	0.69 ± 0.0	−0.17	(−0.23, −0.12)	≤0.001
	Vertical Jump Power (W)					Vertical Jump Power (W/kg)				
10	1339 ± 274	1412 ± 287	−73	(−185, 40)	0.198	36.2 ± 5.6	37.1 ± 5.6	−0.9	(−3.2, 1.3)	0.421
11	1644 ± 48	1486 ± 36	158	(37, 279)	0.011	38.7 ± 0.6	37.6 ± 0.7	1.1	(−0.9, 3.1)	0.263
12	1870 ± 59	1812 ± 73	58	(−134, 252)	0.546	40.2 ± 1.0	40.9 ± 1.1	−0.7	(−3.8, 2,4)	0.668
13	1996 ± 60	2189 ± 82	−193	(−396, 9)	0.074	39.0 ± 0.9	43.3 ± 1.0	−4.3	(−7.2, −1.4)	<0.005
14	2191 ± 55	2772 ± 97	−581	(−804, −358)	≤0.001	40.4 ± 0.7	47.4 ± 1.0	−7.0	(−9.5, 4.6)	≤0.001
15	2194 ± 59	3060 ± 131	−866	(−1154, −577)	≤0.001	40.7 ± 0.7	51.4 ± 1.2	−10.7	(−13.7, −7.7)	≤0.001
16	2409 ± 63	3462 ± 95	−1053	(−1282, −824)	≤0.001	41.6 ± 0.9	52.5 ± 1.1	−10.9	(−13.8, −7.8)	≤0.001
17	2295 ± 66	3567 ± 99	−1272	(−1510, −1034)	≤0.001	40.5 ± 1.0	54.7 ± 1.1	−14.2	(−17.2, −10.8)	≤0.001
18	2437 ± 165	3733 ± 175	−1296	(−1802, −789)	≤0.001	45.1 ± 2.3	58.7 ± 2.5	−13.6	(−20.9, −6.3)	≤0.001

**Figure 3:**
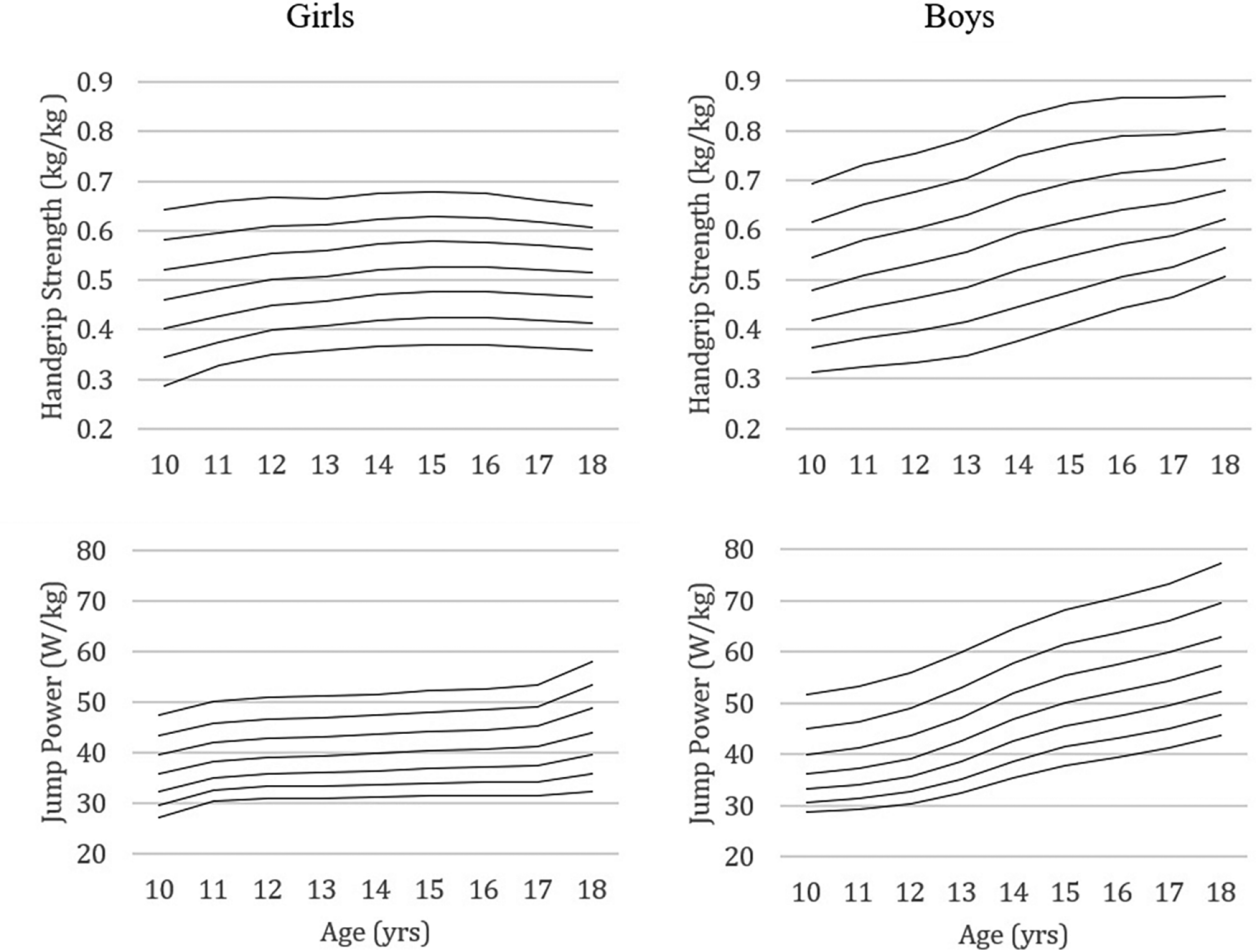
Percentile curves of relative handgrip strength and relative vertical jump power (per kg of body mass) of girls and boys aged 10 to 18.

Table 4 and the corresponding figure (Figure 3) show the results according to the percentile distribution of handgrip strength and relative jumping power with adjustment for body mass. The values of parameters L (lambda), M (mu), and S (sigma) and percentiles 3, 10, 25, 50, 75, 90, and 97 are described according to age and sex. Lambda models depart from normality (skewness); mu represents how musculoskeletal fitness changes with age (median); sigma models the spread of reference values and adjusts for non-uniform dispersion (coefficient of variation).

**Table 4:** L, M, and S parameters and percentile distribution for relative handgrip strength and relative vertical jump power (per kg of body mass) according to sex and age group.

Handgrip strength (kg/kg)																		
	Girls									Boys								
Age	L	S	P3	P10	P25	P50_M	P75	P90	P97	L	S	P3	P10	P 25	P50_M	P75	P90	P97
10	0.89	0.19	0.28	0.34	0.40	0.46	0.52	0.58	0.64	0.31	0.19	0.31	0.36	0.41	0.47	0.54	0.61	0.69
11	0.60	0.17	0.32	0.37	0.42	0.48	0.53	0.59	0.65	0.57	0.19	0.32	0.38	0.44	0.50	0.57	0.65	0.73
12	0.69	0.15	0.35	0.39	0.44	0.50	0.55	0.60	0.66	0.71	0.19	0.33	0.39	0.46	0.53	0.60	0.67	0.75
13	0.83	0.15	0.35	0.40	0.45	0.50	0.55	0.61	0.66	0.77	0.19	0.34	0.41	0.48	0.55	0.62	0.70	0.78
14	1.01	0.14	0.36	0.41	0.46	0.52	0.57	0.62	0.67	0.76	0.19	0.37	0.44	0.51	0.59	0.66	0.74	0.82
15	1.15	0.14	0.36	0.42	0.47	0.52	0.57	0.62	0.67	0.69	0.17	0.40	0.47	0.54	0.61	0.69	0.77	0.85
16	1.17	0.14	0.36	0.42	0.47	0.52	0.57	0.62	0.67	0.65	0.16	0.44	0.50	0.57	0.64	0.71	0.78	0.86
17	1.34	0.14	0.36	0.41	0.47	0.52	0.56	0.61	0.66	0.66	0.15	0.46	0.52	0.58	0.65	0.72	0.79	0.86
18	1.49	0.13	0.35	0.41	0.46	0.51	0.56	0.60	0.65	0.69	0.13	0.50	0.56	0.62	0.68	0.74	0.80	0.86
Vertical jump power (W/kg)																		
Age	L	S	P3	P10	P25	P50_M	P75	P90	P97	L	S	P3	P10	P25	P50_M	P75	P90	P97
10	−0.08	0.14	27.1	29.6	32.3	35.7	39.5	43.3	47.4	−1.41	0.13	28.6	30.6	33.1	36.2	40.0	44.9	51.7
11	−0.53	0.13	30.3	32.6	35.1	38.3	42.0	45.8	50.1	−1.28	0.14	29.1	31.3	33.9	37.1	41.1	46.2	53.1
12	−0.51	0.13	31.0	33.3	35.9	39.1	42.8	46.7	51.0	−1.06	0.14	30.3	32.7	35.7	39.2	43.5	48.9	56.0
13	−0.47	0.13	31.1	33.4	36.0	39.3	43.0	46.8	51.1	−0.72	0.15	32.3	35.2	38.5	42.5	47.2	52.9	59.8
14	−0.34	0.13	31.3	33.7	36.4	39.8	43.5	47.4	51.6	−0.40	0.15	35.2	38.6	42.4	46.8	51.8	57.7	64.5
15	−0.18	0.13	31.5	34.0	36.8	40.3	44.1	48.0	52.2	−0.27	0.14	37.7	41.3	45.4	50.1	55.4	61.4	68.2
16	−0.06	0.13	31.5	34.2	37.1	40.7	44.6	48.5	52.6	−0.24	0.14	39.3	43.1	47.3	52.1	57.5	63.6	70.5
17	0.09	0.14	31.5	34.32	37.4	41.1	45.2	49.2	53.4	−0.27	0.14	41.2	45.0	49.4	54.3	59.8	66.1	73.2
18	0.37	0.15	32.4	35.8	39.6	44.0	48.7	53.3	58.0	−0.38	0.14	43.6	47.6	52.1	57.1	62.9	69.5	77.2

**Figure 4:**
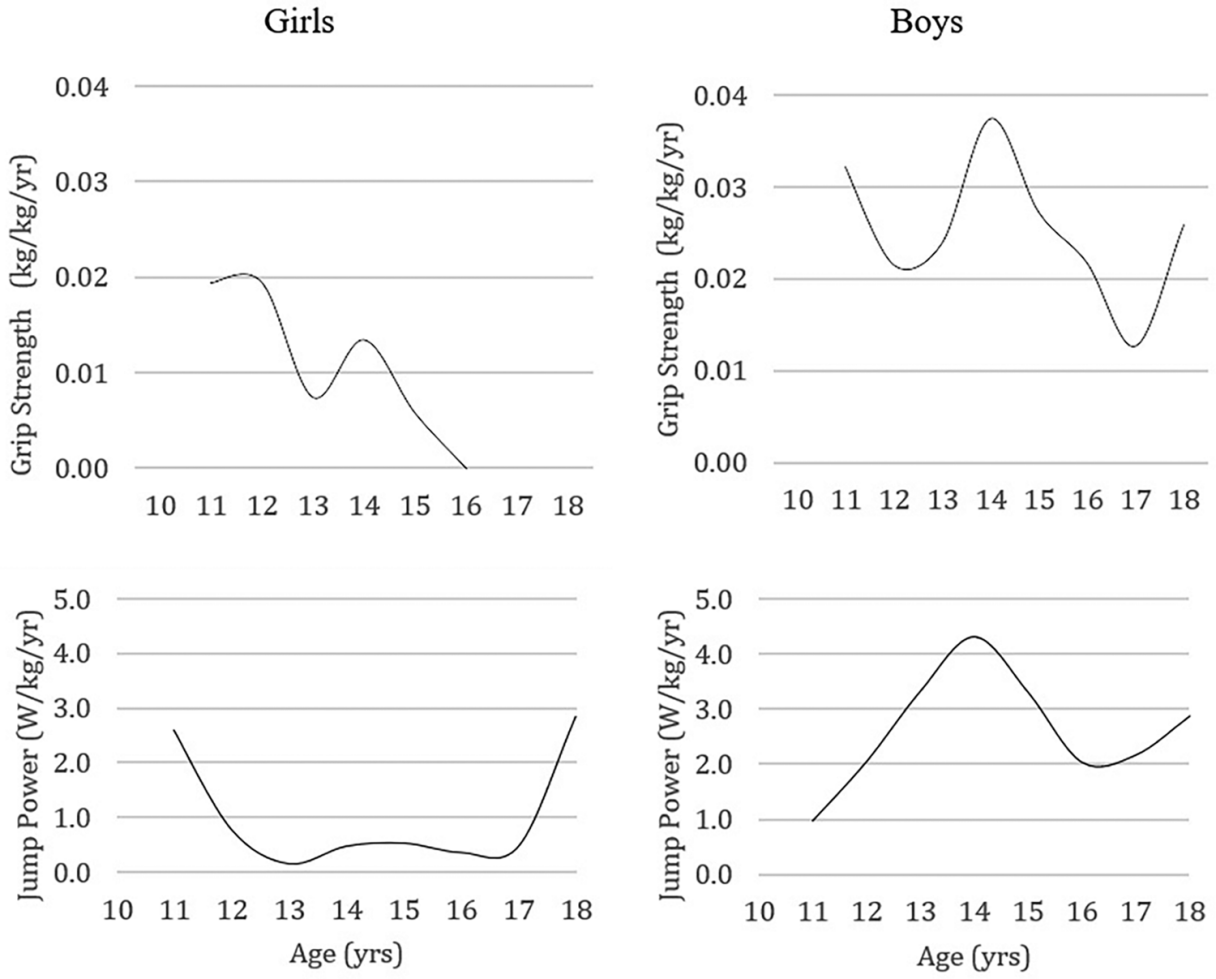
Temporal pattern of development of handgrip strength and vertical jump power per kg of body mass, expressed through the adolescent´s annual gains.

Figure 4 illustrates the estimated annual gains in handgrip strength and jumping power in girls and boys aged 10 to 18. The peak of gains in handgrip strength in girls occurs around 11–12 years (with adjustment for body mass), while in boys, it seems to occur at 14 years. The same trend is observed concerning the peak of gains in vertical jump power in boys, while in girls, the gains seem to be greater at 11 and 18 years old (W/kg).

**Figure 5:**
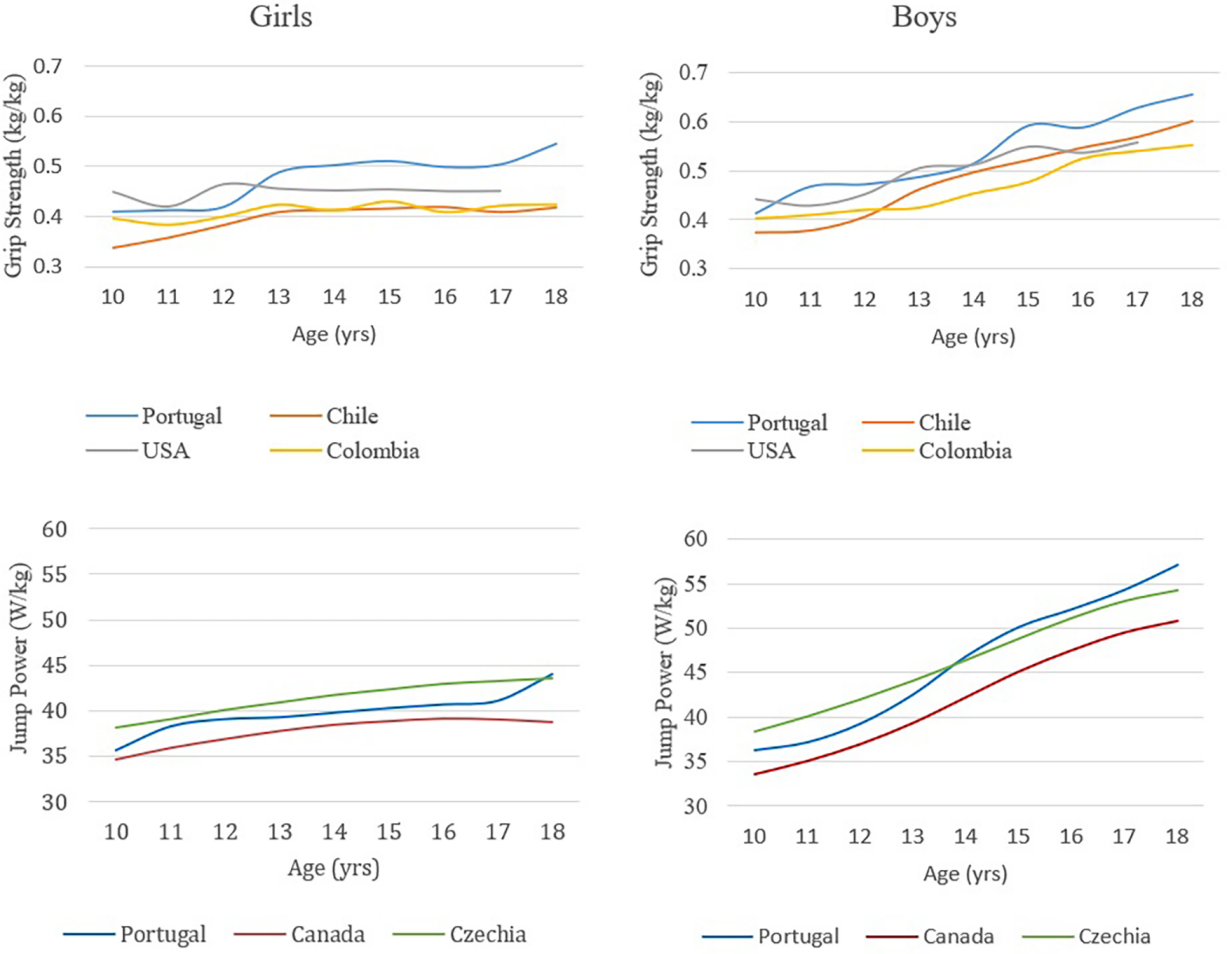
Relative handgrip strength and relative vertical jump power (per kg of body mass) in girls and boys aged 10 to 18. Comparison of the results of this study with the results of studies conducted in Chile (36), the USA (57) and Colombia (35) for handgrip strength and in Canada (45) and the Czech Republic (44) for vertical jump power.

When handgrip strength is adjusted for body mass, Portuguese girls and boys exhibit similar or possibly superior muscle performance compared to other countries with available data for this variable, namely Chile (36), USA (57) and Colombia (35) (Figure 5). Comparative vertical jump power results show relative values (W/kg) similar to or slightly lower than those of the Czech Republic (44) and higher than Canadás (45).

## 4 Discussion

The present study aimed to describe the musculoskeletal fitness of a group of young Portuguese people aged 10–18 years (which previously gave rise to standards for identifying the risk of low muscle mass), investigate sexual differences and compare the musculoskeletal fitness of these young people with those from other countries. For this purpose, handgrip strength at the level of the upper limbs and the power of vertical jump at the level of the lower limbs were considered using procedures similar to those implemented to describe musculoskeletal fitness in young people (9, 32, 33, 35, 36, 45, 47).

Given the importance of musculoskeletal fitness in children and adolescents, whether for detecting talent in the sporting context or identifying risk in the clinical context, it is necessary to have reference standards. Since a specific reference of musculoskeletal fitness depends on the sample values that gave rise to it, it is crucial to portray several samples and investigate dissimilarities between them, preferably considering body size, to mitigate disparities (which also mirror differences in biological maturity) (49). The results obtained in this study are consistent with studies conducted in other countries (9, 32, 33, 35, 36, 45, 47)**,** showing that handgrip strength and vertical jump power increase with age and that boys are generally more robust than girls, especially from 13 to 14 years of age. Sexual dimorphism in musculoskeletal fitness was also observed from these ages in other studies in France (58, 59), the Czech Republic (44), Colombia (47), and Austria (60). For example, Greier and colleagues observed in Austrian adolescents aged 11 to 17 years that, except for flexibility, all other attributes of physical fitness (muscular strength and power, aerobic fitness, agility, balance) remained relatively stable in girls after 13 years of age while in boys continued to increase (60). In Canada, differences in vertical jump power were observed between the sexes from 12 years of age onwards, perhaps because about a third of the sample consisted of Asians, whose maturation occurs earlier than Caucasians (45). Due to an earlier maturation, Asians show better results in vertical jump power than their Caucasian peers. In our sample, the peak in estimated gains in handgrip strength and vertical jump power corresponding to maximal gains (girls: ∼11–12 years; boys: ∼14 years) coincide with the ages at which the differences between the sexes begin to reveal themselves.

Biological and behavioral changes resulting from puberty in girls, namely a greater increase in fat mass and a decrease in physical activity, respectively, may partly explain the sexual dimorphism of physical fitness after puberty; therefore, it is crucial to reinforce interventions to prevent adiposity and promote physical and sports activity in girls (23, 47). However, some studies have observed differences in musculoskeletal fitness (handgrip strength) between the sexes as early as four years of age (26).

Among the different countries with musculoskeletal fitness data for pediatric populations, Chile [4,604 participants (36)] and Colombia [7,268 participants (47)] show absolute values of lower handgrip strength. Body size (body mass, body height) or hand size, in the case of handgrip strength, are usually determinants of these differences (9). When adjusted for body mass, there is a reduction in the differences in handgrip strength between participants with higher body mass (Portuguese and Americans) and those with lower body mass (Chile and Colombia). On the other hand, compared to the USA (57), the results of young people in Portugal seem to improve for girls after 12 years and boys after 14 years.

Regarding vertical jump power, countries with reference standards for this variable generally present similar values, with Canada showing lower values in boys when vertical jump power is adjusted for body mass (Canada: 715 participants (45); Czech Republic: 796 participants (44); Portugal: 736 participants). Although these three studies used the same force platform to assess muscle power (Leonardo Mechanograph), the positioning of hands on the waist (45) or hands-free (44) during the execution of the test may contribute to different results.

Considering the assessment of handgrip strength, previously compared pediatric studies used a dynamometer from the same manufacturer (Jamar, Lafayette, IN, USA), except Ramírez-Vélez and colleagues in Colombia (35) (Takei Scientific Instruments Co., Ltd., Niigata, Japan). Protocol differences verified in body position (standing vs. sitting) or duration of maximum voluntary contraction can also influence performance. The biological maturity of different population groups can also contribute to the diversity of outcomes. For example, Gómez-Campos and colleagues observed significant variability in the biological maturity of Chilean participants, especially during puberty (36). These investigators found that biological age explained more variance in handgrip strength than chronological age. When expressed according to biological age, the handgrip strength of Chilean boys and girls was 5% higher than when expressed according to chronological age. However, there is a trend toward approximating the average handgrip strength in different countries, especially among boys aged 16 and over. In our study, the variance in handgrip strength and vertical jump power adjusted for body mass explained by biological and chronological age was similar: 6% in girls for both ages and 22% vs. 25% in boys in handgrip strength; 8% vs. 9% in girls, and 47% vs. 48% in boys in vertical jump power (data not shown). These are significant associations (*p* < 0.001) but with explanatory variances for biological and chronological age much lower than those observed in our study when handgrip strength and vertical jump power were expressed in absolute values: 49% vs. 58% in girls and 67% vs. 72% in boys in handgrip strength; 43% vs. 51% in girls, and 67% vs. 72% in boys in vertical jump power. This means that adjusting handgrip strength and vertical jump power for body mass limits the influence of maturity on performance in these tests.

The discrepancy in values between countries may also be due to some disparity in the constitution of age groups, every two years in some cases (61, 62), and considering the age range (e.g., 10–11 years for ten years) or average age (e.g., 9.6–10.4 for ten years), although most studies do not describe how they grouped each year of age. The musculoskeletal fitness of our sample was compared with that of other studies with apparently similar age groups (23, 25, 32, 33, 35, 36, 44, 45, 57, 63, 64).

The data from the present study were obtained using the ​LMS method, allowing smoothed curves and a more efficient estimate of the percentiles located at the extremes (36). The percentile curves showed a more or less constant difference in relative handgrip strength between the lower and upper percentiles with advancing age. This observation does not seem corroborated when considering absolute handgrip strength percentile curves (23, 25, 64, 65). Concerning the relative jump power, the percentile curves show some difference with advancing age between the lower and higher percentiles in both sexes.

This work dealt with the most used tests to assess musculoskeletal fitness regardless of age group (young vs. adult vs. older people) and context (clinical and non-clinical). The relevance of the pattern of loss of musculoskeletal fitness during aging is well known, and interest in the pattern of gains during the growing years has only recently emerged. To this end, it is crucial to adjust the results to analyze these patterns throughout the life cycle, which was done in the present study by standardizing for body mass. The published reference for musculoskeletal fitness is based on absolute values ​​that favor heavier people, meaning these people tend to reveal better results (66). This absolute reference does not fail to play a crucial role in the suspicion of musculoskeletal fitness insufficiency. Still, the question remains about the format in which the results of some tests should be expressed (adjusted or not) for the suspicion of outcomes related to body composition, such as sarcopenic obesity, sarcopenic osteopenia, or osteopenic obesity. The main limitations of this work have to do with the sample size and representativeness since it involved a non-population sample based on the convenience of the schools where the participants were recruited for data collection. Although we brought together participants from two studies, the evaluators were the same for each variable. However, intra-observer reproducibility tests were not conducted. Data from other studies reveal strong intra-rater reproducibility with coefficients of variation between 0.1 and 0.3 (55) or correlation coefficients between 0.96 and 0.98 (56). A limitation related to the adjustment variable of musculoskeletal fitness (body mass) can also be considered since other adjustment variables can be used, such as body composition components or body size, and even the format in which they are expressed (e.g., W/kg, W/kg^2/3^). Considering the published standard references, we opted for the most uncomplicated adjustment for body mass.

In conclusion, the present study presents values for handgrip strength and vertical jump power adjusted for body mass, according to chronological age and sex in a group of Portuguese children and young people and their respective trajectories over age between 10 and 18 years, that is, for the period when musculoskeletal fitness develops naturally with growth. Compared to other countries, similar handgrip strength and jumping power values were found in the present study's sample.

## Data Availability

The raw data supporting the conclusions of this article will be made available by the authors, without undue reservation.
